# Advances in Bi_2_WO_6_-Based Photocatalysts for Degradation of Organic Pollutants

**DOI:** 10.3390/molecules27248698

**Published:** 2022-12-08

**Authors:** Haiyan Jiang, Jiahua He, Changyi Deng, Xiaodong Hong, Bing Liang

**Affiliations:** 1 Basic Department, Liaoning Institute of Science and Technology, Benxi 117004, China; 2 School of Materials Science and Hydrogen Energy, Foshan University, Foshan 528000, China; 3 College of Materials Science and Engineering, Shenyang University of Chemical Technology, Shenyang 110142, China

**Keywords:** Bi_2_WO_6_, photocatalysis, degradation performance, composite

## Abstract

With the rapid development of modern industries, water pollution has become an urgent problem that endangers the health of human and wild animals. The photocatalysis technique is considered an environmentally friendly strategy for removing organic pollutants in wastewater. As an important member of Bi-series semiconductors, Bi_2_WO_6_ is widely used for fabricating high-performance photocatalysts. In this review, the recent advances of Bi_2_WO_6_-based photocatalysts are summarized. First, the controllable synthesis, surface modification and heteroatom doping of Bi_2_WO_6_ are introduced. In the respect of Bi_2_WO_6_-based composites, existing Bi_2_WO_6_-containing binary composites are classified into six types, including Bi_2_WO_6_/carbon or MOF composite, Bi_2_WO_6_/g-C_3_N_4_ composite, Bi_2_WO_6_/metal oxides composite, Bi_2_WO_6_/metal sulfides composite, Bi_2_WO_6_/Bi-series composite, and Bi_2_WO_6_/metal tungstates composite. Bi_2_WO_6_-based ternary composites are classified into four types, including Bi_2_WO_6_/g-C_3_N_4_/X, Bi_2_WO_6_/carbon/X, Bi_2_WO_6_/Au or Ag-based materials/X, and Bi_2_WO_6_/Bi-series semiconductors/X. The design, microstructure, and photocatalytic performance of Bi_2_WO_6_-based binary and ternary composites are highlighted. Finally, aimed at the existing problems in Bi_2_WO_6_-based photocatalysts, some solutions and promising research trends are proposed that would provide theoretical and practical guidelines for developing high-performance Bi_2_WO_6_-based photocatalysts.

## 1. Introduction

With the rapid development of various industries, pollution problems, including soil pollution, air pollution, and water pollution, are becoming more and more serious. The problem of water pollution is endangering the health of humans and wild animals; thus, wastewater treatment has become an important task of scientists and technicians. The photocatalysis technique is regarded as an environmentally friendly route for solving water pollution [1]. Therefore, the development of highly active photocatalysts is regarded as the major task of photocatalysis [2]. Lots of semiconductors are adopted as photocatalysts for the degradation of organic pollutants under the irradiation of visible light or UV light [3]. However, the general photocatalytic mechanism of different photocatalysts can be classified into the following three steps: The first step is the generation of photogenerated electrons and holes. When the light irradiation energy surpasses the energy gap (Eg) of the semiconductor, the electrons jump from valence band (VB) to conduction band (CB) and leave holes in the VB [4]. The second step is the transfer of charge carriers. The electrons transfer to the surface of catalysts or recombine with holes. In this process, preventing the recombination of electrons and holes increases the lifetime of photogenerated electrons and holes and enhances the photocatalytic activity by prolonging the redox reaction time [5]. The last step is the surface redox reaction between electrons or holes with O_2_ in H_2_O. Consequently, highly active oxidants are produced and degrade organic pollutants into H_2_O and CO_2_, without secondary pollution. Based on the photocatalytic mechanism, the design principles of high-performance photocatalysts include, but are not limited to, enhancing the light harvesting capability and suppressing the recombination of electrons and holes [6]. Bi_2_WO_6_ is a commonly-used Bi-series semiconductor [7], which has widely served as a visible light photocatalyst for the degradation of organic pollutants due to its advantages of a perovskite-type layered structure, light harvesting ability (band gap of 2.8 eV), low cost, chemical stability, and non-toxicity [8]. In the respect of controllable synthesis, hydrothermal/solvothermal, sol-gel process, calcination, and electrodeposition can be used to prepare Bi_2_WO_6_ nanoplates, nanosheets, nanorods, and nanoflowers or spheres. Besides microstructure control, surface modification and heteroatom doping are adopted for enhancing the photocatalytic activity through generating surface vacancies or defects. In addition, the construction of Bi_2_WO_6_-based composite photocatalysts has become the most popular research topic for the diversity of alternative semiconductive materials.

**Figure 1:**
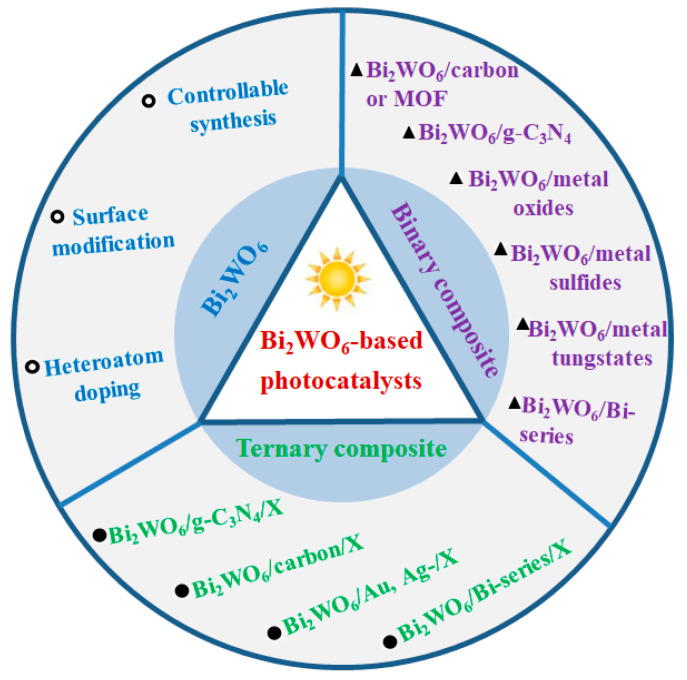
The related research directions of Bi_2_WO_6_-based photocatalysts.

In view of the important role of Bi_2_WO_6_ in Bi-series semiconductor photocatalysts, herein, we summarize the recent advances of Bi_2_WO_6_-based photocatalysts. Figure 1 shows that the whole review involves three sections. The first section is the controllable synthesis, surface modification, and heteroatom doping of Bi_2_WO_6_. In the second section, we majorly introduce the progress of Bi_2_WO_6_-based binary composite photocatalysts, including Bi_2_WO_6_/carbon or MOF composite, Bi_2_WO_6_/g-C_3_N_4_ composite, Bi_2_WO_6_/metal oxides composite, Bi_2_WO_6_/metal sulfides composite, Bi_2_WO_6_/Bi-series composite, and Bi_2_WO_6_/metal tungstates composite. In the last section, Bi_2_WO_6_-based ternary composites are reviewed, including Bi_2_WO_6_/g-C_3_N_4_/X, Bi_2_WO_6_/carbon/X, Bi_2_WO_6_/Au or Ag-based materials/X, and Bi_2_WO_6_/Bi-series semiconductors/X. The design, microstructure, and photocatalytic performance of Bi_2_WO_6_-based binary and ternary composites are highlighted in detail. Lastly, we summarize the research highlights and existing problems in Bi_2_WO_6_-based photocatalysts. Based on the existing problems, some solutions and promising research trends are put forward, finally, that would provide theoretical and practical guidelines for developing novel Bi_2_WO_6_-based photocatalysts.

## 2. Morphology Control, Surface Modification, and Heteroatom Doping of Bi_2_WO_6_

### 2.1. Morphology Control

The microstructure of Bi_2_WO_6_ seriously affects the specific surface area and photocatalytic performance. Therefore, the controllable synthesis and surface modification of Bi_2_WO_6_ have become the basic topic in preparing high-performance Bi_2_WO_6_-based photocatalysts. The synthetic methods of Bi_2_WO_6_ include the hydrothermal/solvothermal method, sol-gel process and calcination, and the electrodeposition method. However, the hydrothermal and solvothermal methods have been widely adopted for fabricating Bi_2_WO_6_, due to the easy operation and controllable microstructure. In this respect, Lai et al. [9] synthesized various Bi_2_WO_6_ photocatalysts via the solvothermal route and discussed the influence of reaction temperature on photocatalytic activity. The BWO-140 sample prepared at 140 °C delivered the best activity for the removal of Erichrome Black T (EBT) dye due to the oxygen vacancies, small size, and large surface area. Selvi et al. [10] discussed the influence of reaction time on the performance of Bi_2_WO_6_ photocatalysts. Under the same hydrothermal conditions, the nanoplates of Bi_2_WO_6_-24 h delivered the optimum photocatalytic activity for the degradation of MB due to the narrow band, smaller crystallite size, and hierarchical structure.

### 2.2. Surface Modification

Besides the effect of reaction conditions, hexadecyl trimethyl ammonium bromide (CTAB) and polyvinylpyrrolidone (PVP) are adopted to adjust the microstructure of Bi_2_WO_6_. By using CTAB and PVP surfactants, Guo et al. [11] prepared nanosheet-assembled Bi_2_WO_6_ microspheres and investigated the growth mechanism of the assembled microspheres. When used for the degradation of Rh B under visible light, the degradation efficiency was 98% within 50 min. By adopting CTAB surfactant and the mixed solvent of ethyl alcohol and ethylene glycol, Bai et al. [12] synthesized Bi_2_WO_6_ photocatalysts with abundant oxygen vacancies. The generation of oxygen vacancy enhanced the photogenerated carrier separation efficiency and visible light absorption ability, which resulted in superior photocatalytic activity for the degradation of ciprofloxacin, and the degradation rate reached 90% within 6 h. In addition, the CTAB-capped Bi_2_WO_6_ photocatalyst was synthesized with flower-like structures [13]. The CTAB surfactant affected the microstructure, which facilitated the physical adsorption of Rh B dye and enhanced the photoactivity of the resulting product. Consequently, the 0.20CTAB-Bi_2_WO_6_ sample degraded 100% Rh B within 120 min. By using the CTAB surfactant and an ethylene glycol-water mixed solvent, Zhou et al. [14] synthesized Bi_2_WO_6_ and Au-decorated Bi_2_WO_6_ hollow microspheres. When used for the degradation of phenol under visible light, Au nanoparticle-decorated Bi_2_WO_6_ delivered enhanced photocatalytic activity due to the cooperative electron trapping abilities and the SPR effect of Au nanoparticles.

### 2.3. Heteroatom Doping

Heteroatom doping can be adopted to adjust the crystal plane structure of Bi_2_WO_6_ and broaden the light absorption range, which is widely reported to enhance the photoactivity of Bi_2_WO_6_. In this section, we summarize the advances of non-metal-doped Bi_2_WO_6_ and metal-doped Bi_2_WO_6_.

In the existing non-metal dopants, N, F, Cl, and I serve as dopants for fabricating non-metal-doped Bi_2_WO_6_ photocatalysts, and the major topics mostly involve the influence of the doping amount on the microstructures and photodegradation performance. In this respect, Hoang et al. [15] synthesized N-doped Bi_2_WO_6_ nanoparticles and discussed the influence of the N-doping amount on photocatalytic activity. Through a comparison, the doped sample with a N/Bi atomic ratio of 0.5% presented the best photodegradation performance for removing Rh B under the irradiation of visible light. Chen et al. [16] prepared F-doped Bi_2_WO_6_ via a hydrothermal route and investigated the effect of the F-doping amount on the sample morphology and degradation performance. With an increase of the F-doping amount, ultrathin Bi_2_WO_6_ nanosheets were transformed into hierarchical nanoflowers. When used for the degradation of tetracycline (TC), the degradation rate constant of the optimized F-BWO4 sample was about 4.5 times higher than that of pristine Bi_2_WO_6_ due to the hierarchical structure and strong electronegativity. Phuruangrat et al. [17] reported the preparation of I-doped Bi_2_WO_6_ photocatalysts and their degradation performance. The results showed that 3 wt% I-doped Bi_2_WO_6_ presented the best performance, which degraded 100% Rh B in 100 min under the radiation of visible light, with a degradation rate of 0.044 min^−1^.

**Figure 2:**
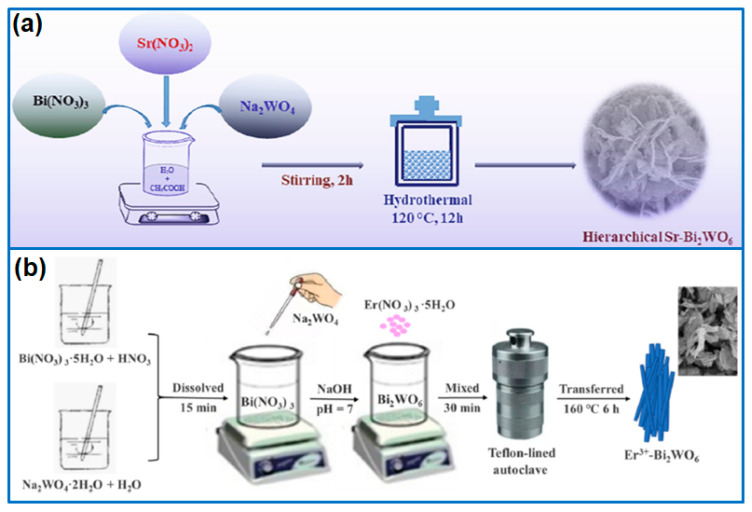
Fabrication process of (**a**) hierarchical Sr-Bi_2_WO_6_ [20]. Copyright (2022) Elsevier. (**b**) Er^3+^-Bi_2_WO_6_ photocatalyst [21]. Copyright (2022) Elsevier.

Compared to non-metal doping, there are abundant works about metal-doped Bi_2_WO_6_, including Fe, Ti, Sr, Er, La, Au, Ag, and Mo dopants. Besides mono-metal doping, dual-metal doping was reported to further improve the photocatalytic activity of Bi_2_WO_6_. Among various metal dopants, the incorporation of Fe dopants would accelerate the electron-hole separation and improve the photocatalytic activity. In respect of the Fe-doping mechanism, Hu et al. [18] confirmed that Fe doping narrowed the energy band gap and induced abundant oxygen vacancies, which enhanced the separation efficiency of photogenerated carriers and the light absorption capability. When used for the degradation of Rh B and salicylic acid (SA), the optimized BW-Fe-0.10 sample showed 11.9 and 8.0 times higher than that of pristine Bi_2_WO_6_, respectively. Arif et al. [19] prepared Ti-doped Bi_2_WO_6_ photocatalysts and confirmed that the presence of Ti^3+^/Ti^4+^ in Ti-doped Bi_2_WO_6_ promoted the generation of reactive oxygen species, which greatly enhanced the photocatalytic activity of Bi_2_WO_6_. Furthermore, the layered 3D hierarchical structure adjusted the band structure of Bi_2_WO_6_, further facilitating the enhancement of the photocatalytic performance. Maniyazagan et al. [20] synthesized hierarchical Sr-Bi_2_WO_6_ photocatalysts for the degradation of 4-NP and MB. As shown in Figure 2a, by optimizing the content of Sr^2+^ ions, the composite of 15% Sr-Bi_2_WO_6_ delivered the highest photocatalytic activity. Under the irradiation of UV light with NaBH_4_, the optimized sample degraded 99.5% MB in 25 min and 99.4% 4-NP reduction in 15 min, respectively. The major reason was the enhanced charge carrier separation and the generation of oxygen vacancies. Qiu et al. [21] fabricated an Er^3+^-mixed Bi_2_WO_6_ photocatalyst by a one-step hydrothermal route (Figure 2b). After adding Er^3+^ ions, Bi_2_WO_6_ was transformed into a layered nanosheet with a high specific surface area. The sample of 16% Er^3+^-Bi_2_WO_6_ presented a high degradation rate of 94.58% TC within 60 min. The enhanced activity was attributed to the porous structure and enhanced separation efficiency of photogenerated electrons. Ning et al. [22] synthesized La^3+^-doped Bi_2_WO_6_ nanoplates for the degradation of Rh B. Compared to Bi_2_WO_6_, the doped sample showed a higher specific surface area, and the band gap reduced to 2.81 eV from 2.89 eV. Furthermore, the La^3+^-doping enhanced the separation efficiency of electron and hole pairs. Therefore, the La^3+^-doped Bi_2_WO_6_ presented a higher degradation rate constant than pure Bi_2_WO_6_.

In respect of noble metal dopants, Phuruangrat et al. [23] synthesized Au-doped Bi_2_WO_6_ and incorporated Au^3+^ ions into Bi_2_WO_6_ lattice. By adjusting the doping amount of Au, 3% Au-doped Bi_2_WO_6_ nanoplates presented the highest Rh B degradation rate of 96.25% within 240 min, which was 2.15 times higher than that of pure Bi_2_WO_6_. The superior performance was ascribed to the enhanced separation efficiency of photogenerated electrons and holes. In another work, Phu et al. [24] discussed the photocatalytic activities of Ag-doped Bi_2_WO_6_ and Ag nanoparticle-decorated Bi_2_WO_6_. For Ag-doped sample, Ag ions substituted the lattice of Bi_2_WO_6_, while, for the decorated sample, abundant Ag nanoparticles were dispersed on the surface of Bi_2_WO_6_ nanoparticles with no lattice change. When used for the degradation of Rh B by visible light, the activity of the Ag nanoparticles-modified sample was more than two times higher than that of the Ag-doped sample due to the enhanced surface plasmon resonance caused by Ag nanoparticles. Besides mono-metal doping, (La, Mo) co-doped Bi_2_WO_6_ was reported [25]. The introduction of La and Mo adjusted the particle size and lattice spacing of Bi_2_WO_6_. Moreover, the La and Mo co-dopants inhibited the charge recombination. Consequently, the (0.25La, 0.25Mo)-Bi_2_WO_6_ sample containing 0.25 mol% La and 0.25 mol% Mo showed the highest activity for the photodegradation of MB.

## 3. Bi_2_WO_6_-Based Binary Composite

Besides the decoration or doping of Bi_2_WO_6_, the construction of Bi_2_WO_6_-based composite is widely reported for enhancing the photoactivity of Bi_2_WO_6_. According to the type of candidate materials, we classified the existing Bi_2_WO_6_-based binary composites into six types: Bi_2_WO_6_/carbon or MOF composite, Bi_2_WO_6_/g-C_3_N_4_ composite, Bi_2_WO_6_/metal oxides composite, Bi_2_WO_6_/metal sulfides composite, Bi_2_WO_6_/Bi-series composite, and Bi_2_WO_6_/metal tungstates composite. The design idea, microstructure, and photocatalytic performance of these binary composite photocatalysts are summarized in detail.

### 3.1. Bi_2_WO_6_/Carbon or MOF Composite

Carbon materials exhibit good conductivity and a large specific surface area, which are more suitable for loading Bi_2_WO_6_ nanostructures. In this section, various carbon materials, including graphene, carbon nanotube, carbon dots, and biomass-derived carbon are used for hybridizing with Bi_2_WO_6_. Among carbon materials, graphene oxide (GO) is a typical two-dimension template with abundant oxygen-containing groups, which can be served as ideal 2D substrates for loading semiconductors. Compared to GO, reduced GO (rGO) exhibits a superior electronic conductivity, which was adopted to hybridize with Bi_2_WO_6_ to enhance the photodegradation efficiency. For example, Zhao et al. [26] fabricated an rGO/Bi_2_WO_6_ composite photocatalyst via the hydrothermal method, and the composite degraded 87.49% norfloxacin within 180 min. The photocatalytic activity was much higher than that of pure Bi_2_WO_6_ due to the efficient charge separation and enhanced light-harvesting capacity. Arya et al. [27] also prepared an rGO-Bi_2_WO_6_ heterostructure via hydrothermal route and investigated their photocatalytic activity for the removal of levofloxacin. When kept in visible light at room temperature, the rGO-Bi_2_WO_6_ composite achieved a high degradation rate of 74.3% within 120 min due to the inhibition of charge carrier recombination. Furthermore, multiwalled carbon nanotubes (MWNTs) were coupled with Bi_2_WO_6_ to fabricate 3D mesoporous MWNTs-Bi_2_WO_6_ microspheres [28]. The MWNTs promoted the transfer and separation of hole and electron pairs, which enhanced the light absorption capability of Bi_2_WO_6_. The composite containing 3% MWNTs showed the optimum photoactivity, and the degradation efficiency was 1.35 times higher than that of pure Bi_2_WO_6_. In addition, carbon dots (CDs) were used for decorating a 3D Cl-doped Bi_2_WO_6_ hollow microsphere to construct CDs/Cl-Bi_2_WO_6_ composite photocatalysts [29]. The introduced CDs and Cl doping enhanced the visible light absorption capability and inhibited the recombination of electron–hole pairs. The optimized 0.5% CDs/Cl-Bi_2_WO_6_ composite degraded 85.1% TCH within 60 min, which was much better than Bi_2_WO_6_ and Cl-Bi_2_WO_6_.

**Figure 3:**
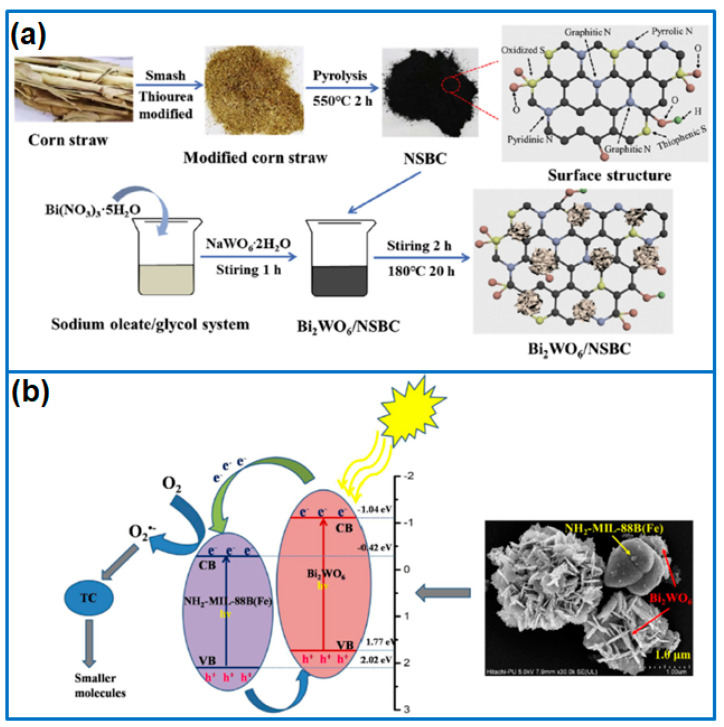
Preparation of (**a**) Bi_2_WO_6_/NSBC composite [32]. Copyright (2021) Elsevier. (**b**) Bi_2_WO_6_/NH_2_-MIL-88B (Fe) heterostructure [33]. Copyright (2021) Elsevier.

In addition, biomass-derived carbon materials were coupled with Bi_2_WO_6_ to fabricate Bi_2_WO_6_/C hybrid photocatalysts. In this respect, Liang et al. [30] firstly prepared bamboo leave-derived carbon and then fabricated 3D flower-like Bi_2_WO_6_/C composites by hydrothermal route. The large specific surface area of biomass carbon enhanced the adsorption capacity; meanwhile, the good conductivity promoted the separation of charge carriers. The optimized Bi_2_WO_6_/C (6: 1) sample had a high degradation rate of 85.4% for the removal of TC within 90 min. Wang et al. [31] prepared a Bi_2_WO_6_/N-modified biochar (BW/N-B) composite for the degradation of Rh B pollutant. The loading of N-B improved the photocatalytic activity due to the enhanced separation and transfer of electron–hole pairs. Under the irradiation of visible light, the BW/N1-B sample with a urea/biochar ratio of 2:1 presented the best photocatalytic activity, which degraded 99.1% Rh B within 45 min. In addition, N and S co-doped corn straw biochar (NSBC) was used to hybridize with Bi_2_WO_6_ to form Bi_2_WO_6_/NSBC composite photocatalysts (Figure 3a) for the degradation of ciprofloxacin (CIP) [32]. The N and S co-doped biochar exhibited a high specific surface area and interconnected fiber structure and high catalytic property, which effectively prevented the agglomeration of Bi_2_WO_6_. The combination of NSBC and Bi_2_WO_6_ extended the visible light response, adjusted the band gap, and promoted the separation and transfer of photoinduced carriers, which achieved the fast degradation of CIP (5 mg/L) within 75 min.

Metal-organic frameworks (MOFs) consist of metal ions or metal clusters and organic ligands, which show some advantages in permanent porosity, tunable pore size, high specific surface area, and active surface chemistry. Various MOFs are widely utilized in adsorption, gas storage and separation, energy storage, and photocatalysis. In the field of photocatalysis, various MOFs, including Fe-based MOFs and Zn-based MOFs, are coupled with Bi_2_WO_6_ to fabricate hybrid photocatalysts. In this respect, MIL-100(Fe) nanoparticles were hybridized with Bi_2_WO_6_ nanosheets to construct MIL-100(Fe)/Bi_2_WO_6_ Z-scheme heterojunction [34]. When tested for the degradation of TC under sunlight, the optimized 12%MIL/BWO delivered the highest photocatalytic activity due to the large specific surface area, enhanced light adsorption range, and high charge transfer and separation efficiency. In addition, the MIL-88A(Fe)/Bi_2_WO_6_ heterojunction was synthesized for the degradation of Rh B and TC [35]. The combination of MIL-88A(Fe) and Bi_2_WO_6_ effectively inhibited the recombination of photogenerated carriers. Under the irradiation of visible light, the heterojunction degraded 96% Rh B and 71% TC within 50 min and 80 min, respectively. Kaur et al. [33] prepared a Bi_2_WO_6_/NH_2_-MIL-88B(Fe) heterostructure for the degradation of TC (Figure 3b). Due to the promoted separation and transfer of photoexcited charges caused by the interfacial contact, the heterostructure presented a high degradation efficiency of 89.4% within 130 min under solar illumination. Tu et al. [36] synthesized MIL-53(Fe)/Bi_2_WO_6_ heterostructure photocatalysts. The formation of heterojunction extended the visible light absorption capability and accelerated the transfer of photogenerated electrons. When used for the degradation of Rh B and phenol Rh B under visible light irradiation, the degradation rate constants of the heterostructure containing 5 wt% MIL-53(Fe) were 3.75-fold and 3.27-fold higher than that of pristine Bi_2_WO_6_.

Besides various Fe-based MOFs, Zhang et al. [37] prepared hydrangea-like Bi_2_WO_6_/ZIF-8 (BWOZ) hybrid photocatalysts by using a flower-like Bi_2_WO_6_ template. The generation of heterojunction induced the fast separation of photogenerated carriers. Meanwhile, the optimized BWOZ containing 7.0 wt% Bi_2_WO_6_ showed a large specific surface area, which degraded 85.7% MB within 240 min, and the reaction kinetic constant was 23-fold and 1.61-fold higher than that of pure Bi_2_WO_6_ and ZIF-8, respectively. In addition, Dai et al. [38] also fabricated Bi_2_WO_6_/ZIF-8 composite photocatalysts for the degradation of TC. Under the irradiation of UV light, the optimized sample achieved the fast degradation of 97.8% TC (20 mg/L) within 80 min, and the degradation rate constant was about 3-fold higher than that of pure Bi_2_WO_6_.

### 3.2. Bi_2_WO_6_/g-C_3_N_4_ Composite

**Figure 4:**
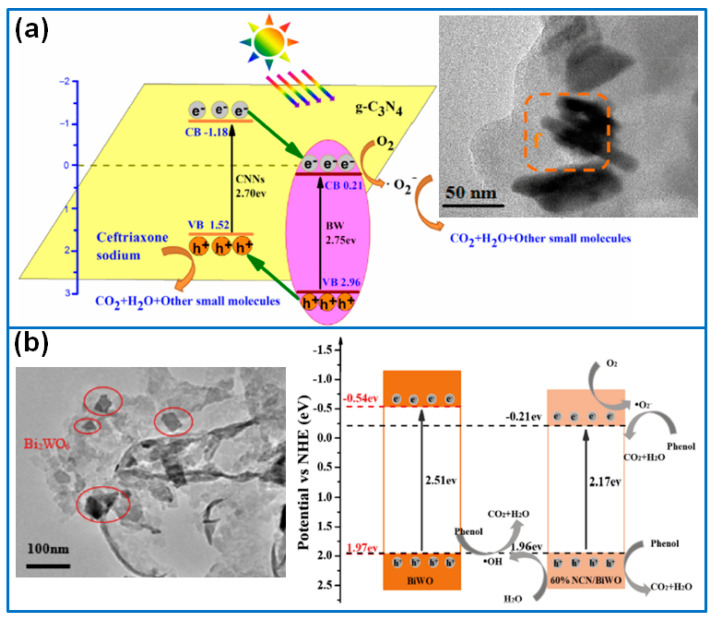
Photocatalytic mechanism of (**a**) Bi_2_WO_6_/g-C_3_N_4_ [40]. Copyright (2018) Elsevier. (**b**) N-doped g-C_3_N_4_ (NCN)/Bi_2_WO_6_ [42]. Copyright (2020) Elsevier.

Serving as a non-metal semiconductor, graphitic carbon nitride (g-C_3_N_4_) presents two-dimensional graphite-like structures with a high specific surface area, which are widely adopted as active templates for loading various semiconductor photocatalysts. In this section, we introduce the research progress of Bi_2_WO_6_/g-C_3_N_4_ composite photocatalysts. Qi et al. [39] prepared Bi_2_WO_6_/g-C_3_N_4_ heterojunction by hydrothermal reaction and discussed the effect of g-C_3_N_4_/Bi_2_WO_6_ ratio on photodegradation performance. When the Bi_2_WO_6_ ratio was 10 wt%, the composite presented the highest photocatalytic activity for the degradation of MB, and the reaction rate constant was 4 and 1.94 times higher than that of pristine g-C_3_N_4_ and Bi_2_WO_6_, respectively. In addition, Zhao et al. [40] also prepared Bi_2_WO_6_/g-C_3_N_4_ (BW/CNNs, Figure 4a) composite photocatalysts for the degradation of Ceftriaxone sodium in an aquatic environment. The combination of the two components enhanced the absorption capability of visible light and accelerated the separation of photogenerated electron–hole pairs. As a result, the 40%-BW/CNNs delivered the best photocatalytic activity, which degraded 94.50% Ceftriaxone sodium within 120 min under the irradiation of visible light. Chen et al. [41] synthesized Bi_2_WO_6_ on g-C_3_N_4_ to fabricate Bi_2_WO_6_/g-C_3_N_4_ heterojunctions by a hydrothermal route. When used for the degradation of Rh B and phenol solution, the composite with a Bi/g-C_3_N_4_ molar ratio of 4% presented the highest photodegradation activity for the highest separation efficiency of photogenerated electron–hole pairs. Moreover, the separation and transfer of electron–hole pairs were proven as a direct Z-scheme mechanism.

In respect of the microstructure design, Zhu et al. [42] prepared various N-doped g-C_3_N_4_ (NCN)/Bi_2_WO_6_ (BWO) composites by using NCN as the template, as shown in Figure 4b. The ratio of NCN/BWO was adjusted to reduce the band gap and increase the surface area. The 60% NCN/BWO presented the best photocatalytic activity, which achieved the fast degradation of 93.1% phenol within 5 h, and the degradation rate constant was 18.5 times higher than that of Bi_2_WO_6_. The major reason was ascribed to the enhanced visible light absorption capability caused by NCN. Wang et al. [43] prepared core-shell structured g-C_3_N_4_@Bi_2_WO_6_ composite by in situ, forming an ultrathin g-C_3_N_4_ shell layer on Bi_2_WO_6_ nanosheets. By adjusting the thickness of the g-C_3_N_4_ shell layer, the interface of g-C_3_N_4_@Bi_2_WO_6_ was optimized to promote the separation efficiency of photogenerated electron–hole pairs. As a result, the composite photocatalyst with a 1 nm-thick shell layer delivered the optimum degradation phenol activity under visible light, which was about 1.9 times higher than that of Bi_2_WO_6_ and 5.7 times higher than that of g-C_3_N_4_. Zhang et al. [44] prepared g-C_3_N_4_ quantum dot (CNQD)-decorated ultrathin Bi_2_WO_6_ nanosheets for the degradation of Rh B and tetracycline (TC) under the irradiation of visible light. By optimizing the ratio, the composite of 5% CNQDs/BWO delivered the highest photocatalytic performance, which degraded 87% TC and 92.51% Rh B within 60 min. The superior activity can be ascribed to the Z-scheme charge transfer mechanism, the up-conversion behavior of CNQDs, and the enhanced separation and transfer rates of photo-generated charges. Regarding the performance of different S-scheme heterojunctions, Gordanshekan et al. [45] compared the photocatalytic activity of Bi_2_WO_6_/g-C_3_N_4_ with Bi_2_WO_6_/TiO_2_. When serving as photocatalysts for removing cefixime (CFX) in polluted water, Bi_2_WO_6_/g-C_3_N_4_ showed a removal efficiency of 94%, which was slightly higher than that of Bi_2_WO_6_/TiO_2_ (91%).

### 3.3. Bi_2_WO_6_/Metal Oxides Composite

Metal oxides have a wide range, and most of them can serve as semiconductor photocatalysts. To further enhance the photoactivity of Bi_2_WO_6_, various metal oxide semiconductors, including TiO_2_, ZnO, and other metal oxides, are reported to hybridize with Bi_2_WO_6_, and the research progress of Bi_2_WO_6_/metal oxides composites is introduced in this section.

#### 3.3.1. Bi_2_WO_6_/TiO_2_ Composite

Compared to other metal oxides, TiO_2_ exhibits some advantages of low cost, low toxicity, strong redox ability, and high catalytic activity, and it is regarded as the most popular photocatalyst in the past few decades. However, the wide band gap of 3.0~3.2 eV limits the visible light adsorption, only allowing a UV light response. Moreover, the fast recombination of photogenerated electron–hole pairs further weakens the photocatalytic activity of TiO_2_. The combination of TiO_2_ with Bi_2_WO_6_ overcomes the shortage of TiO_2_, and various TiO_2_/Bi_2_WO_6_ composite photocatalysts were developed in recent years. For example, Li et al. [46] fabricated TiO_2_/Bi_2_WO_6_ microflowers via a one-step hydrothermal reaction, in which TiO_2_ nanoparticles (10 nm) were dispersed on Bi_2_WO_6_ microflowers. The TiO_2_/Bi_2_WO_6_ composite degraded 100% Rh B within 60 min under visible light or 30 min under UV-vis light. The enhanced photocatalytic activity was ascribed to the synergistic effect of two components. Furthermore, the improved light adsorption capacity and carrier separation efficiency also facilitated the photoactivity. In addition, an electrospinning technique was adopted to fabricate Bi_2_WO_6_/TiO_2_ nanofibers (BTNF) by decorating Bi_2_WO_6_ nanosheets on the TiO_2_ fiber surface [47]. In this composite, the Bi_2_WO_6_ extended the light absorption range, and the Bi_2_WO_6_/TiO_2_ heterojunction promoted the charge separation. Therefore, BTNF presented a superior visible light activity for degrading Rh B, which was much better than pure Bi_2_WO_6_, TiO_2_, and their mixture. Lu et al. [48] deposited Bi_2_WO_6_ nanosheets onto TiO_2_ nanotube arrays (TNTAs) to fabricate BWO/TNTAs composite photocatalysts. The 0.2BWO/TNTAs sample achieved the fast degradation of 92.2% TC within 180 min due to the promoted charge separation and extended light absorption range.

Besides the direct combination of Bi_2_WO_6_ and TiO_2_, Wang et al. [49] prepared Sb^3+^ doped Bi_2_WO_6_/TiO_2_ nanotube photocatalysts. A rose-like BWO-10 sample showed superior photocatalytic activity, which achieved the fast degradation of 80.58% Rh B, 77.23% MO, and 99.06% MB under the irradiation of visible light, and the best performance resulted from the uniform rose-like structure and adjusted energy level. Sun et al. [50] prepared N/Ti^3+^ co-doped TiO_2_/Bi_2_WO_6_ heterojunctions (NT-TBWx) and proved that the degradation rate order of organic pollutants was photocatalysis < sonocatalysis < sonophotocatalysis. The superior performance was attributed to the doping level, heterophase junction, and heterojunction.

#### 3.3.2. Bi_2_WO_6_/ZnO Composite

To achieve the combination of narrow-band gap Bi_2_WO_6_ and wide-band gap ZnO, various ZnO/Bi_2_WO_6_ (ZBW) heterostructures were developed to improve the photocatalytic activity of Bi_2_WO_6_. In this respect, Liu et al. [51] synthesized the ZBW heterostructure via the hydrothermal method and investigated the photocatalytic activity for degrading MB under ultraviolet light. ZBW degraded 95.48% MB within 120 min, and the excellent photocatalytic performance was due to the promoted separation of electrons and holes caused by the heterojunction. Koutavarapu et al. [52] synthesized a hetero-structured Bi_2_WO_6_/ZnO composite via a hydrothermal route for the degradation of Rh B under solar irradiation. The formation of a Bi_2_WO_6_/ZnO interface reduced the charge transfer resistance and inhibited the recombination of charge carriers. By adjusting the additional amount of ZnO, the optimized BWZ-20 composite showed the best photocatalytic activity, which degraded 99% Rh B within 50 min. Chen et al. [53] synthesized a ZnO/Bi_2_WO_6_ heterostructure on flexible carbon cloth (CC) substrate. The optimized Z3B-CC sample containing 3 wt% Bi_2_WO_6_ degraded 96.9% MB within 100 min, which also facilitated the reuse of the photocatalyst. The enhanced activity was related to the enhanced light absorption range and the formation of a type-II energy band structure. To further enhance the activity, Zhao et al. [54] prepared a Z-scheme C and N-co-doped ZnO/Bi_2_WO_6_ (CZB) hybrid photocatalyst, and the influence of the C and N-co-doped ZnO content on the photodegradation performance of CZB composites was investigated. In this complicated structure, C and N co-doping adjusted the energy level and enhanced light absorption. Furthermore, residual C accelerated the separation and transfer of photogenerated carries. Through a comparison, CZB containing 5 wt% C/N-ZnO presented the best activity for the removal of tetracycline, enrofloxacin, and norfloxacin under visible light, and the photodegradation mechanism was confirmed as the formation of a Z-scheme heterojunction.

#### 3.3.3. Bi_2_WO_6_/Other Metal Oxides Composite

**Figure 5:**
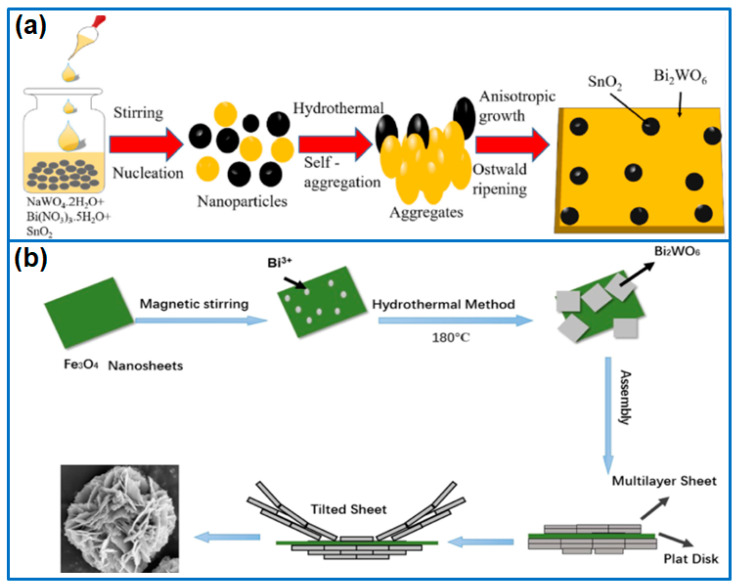
Fabrication process of (**a**) SnO_2_/2D-Bi_2_WO_6_ [56]. Copyright (2021) Elsevier. (**b**) Fe_3_O_4_/Bi_2_WO_6_ heterojunction [57]. Copyright (2021) American Chemical Society.

Besides commonly used TiO_2_ and ZnO, other metal oxides, including SnO_2_, MnO_2_, Co_3_O_4_, Fe_3_O_4_, CuO, WO_3_, Bi_2_O_4_, and In_2_O_3_, were hybridized with Bi_2_WO_6_ to enhance photocatalytic activity. For example, Salari et al. [55] prepared Z-scheme flower-like Bi_2_WO_6_/MnO_2_ composite photocatalysts, in which MnO_2_ nanoparticles that were dispersed on 3D Bi_2_WO_6_ flowers enhanced the transfer and separation of charge carriers. As a result, the optimized Bi_2_WO_6_/MnO_2_ (1:10) degraded 100% MB within 100 min. Mallikarjuna et al. [56] deposited small SnO_2_ nanoparticles onto Bi_2_WO_6_ nanoplates to fabricate SnO_2_/2D-Bi_2_WO_6_ photocatalysts (Figure 5a). The loading of SnO_2_ nanoparticles adjusted the visible light absorption region and promoted charge separation and transfer efficiency. When used for degrading the Rh B pollutant, the photocatalytic activity of the composite was more than 2.7 times higher than that of 2D-Bi_2_WO_6_ nanoplates.

Zhang et al. [58] prepared flower-like Co_3_O_4_ QDs/Bi_2_WO_6_ composite photocatalysts to achieve the uniform dispersion of Co_3_O_4_ QDs on flower-like nanosheets. By adjusting the ratio of Co_3_O_4_ QDs, the sample of 10%-Co_3_O_4_ QDs/Bi_2_WO_6_ presented the optimum performance for the removal of TC, and the degradation rate constant was about 1.55 and 3.40 times higher than that of Bi_2_WO_6_ and Co_3_O_4_ QDs, respectively. The superior performance was ascribed to the formation of a p–n heterojunction and enhanced the visible light absorption capacity. Zhu et al. [57] prepared Z-scheme Fe_3_O_4_/Bi_2_WO_6_ heterojunctions as photocatalysts for degrading ciprofloxacin (CIP), in which the flower-like composite was assembled by abundant nanosheets (Figure 5b). The formation of Z-scheme heterojunctions facilitated the light-harvesting capacity and suppressed the recombination of photogenerated carriers. Under the irradiation of visible light, the FB-180 sample prepared at 180 °C with 4% Fe delivered optimum photoactivity, which degraded about 99.7% CIP within 15 min. Moreover, the sample showed superior reusability and stability. Koutavarapu et al. [59] fabricated CuO/Bi_2_WO_6_ (CuBW) composite photocatalysts for degrading TC and MB. In this composite, Bi_2_WO_6_ provided transfer pathways for photogenerated electrons, while CuO was used to receive carriers from Bi_2_WO_6_ and inhibited the recombination of charge carriers; thus, the formation of the heterostructure improved the photocatalytic activity. The optimized CuBW sample containing 10 mg Bi_2_WO_6_ showed the highest degradation efficiency, which degraded 97.72% TC within 75 min and 99.43% MB within 45 min.

In respect of interface engineering design, Chen et al. [60] achieved the in-situ growth of (001)- and (110)-exposed WO_3_ on (010)-exposed Bi_2_WO_6_ to form Z-scheme heterojunction photocatalysts. The facet control produced some dislocation defects for promoting the carriers transfer. Furthermore, Z-scheme transfer mode optimized the transfer of photogenerated electrons and improved the oxidization ability of photogenerated holes. Consequently, the WO_3_ (001) and (110)/Bi_2_WO_6_ achieved a high removal rate of 74.5% for salicylic acid within 6 h, and the kinetic constant was 2.4 times higher than that of WO_3_ (001)/Bi_2_WO_6_.

In addition, Bi_2_O_4_ micro-rods were in situ grown on Bi_2_WO_6_ microspheres to form a Bi_2_O_4_/Bi_2_WO_6_ heterojunction [61]. The heterojunction facilitated the separation and transfer of charge carriers. When used for the degradation of Rh B under visible light, the degradation rate constant of the composite was 5 times higher than that of pure Bi_2_WO_6_. While degrading MO, the enhanced factor reached 90-fold. Besides Bi_2_O_4_, a flower-like Bi_2_WO_6_/Bi_2_O_3_ photocatalyst was also synthesized by the ionic liquid solvothermal method and calcination [62], and it presented a higher photocatalytic H_2_ production activity than pure Bi_2_WO_6_. Qin et al. [63] prepared a rich oxygen vacancy (OVs) Bi_2_WO_6_/In_2_O_3_ hybrid photocatalyst for the degradation of Rh B. The formation of a Bi_2_WO_6_/In_2_O_3_ heterostructure extended the lifetime of photogenerated charge carriers. Furthermore, the OVs in Bi_2_WO_6_/In_2_O_3_ accelerated the separation of photogenerated electron–hole pairs. Through comparison, the BiIn80 sample containing 80 wt% Bi_2_WO_6_ exhibited the best photocatalytic activity, and the reaction rate constant was about 4.17-fold and 15-fold higher than that of Bi_2_WO_6_ and In_2_O_3_, respectively.

### 3.4. Bi_2_WO_6_/Metal Sulfides Composite

To effectively optimize the band edge of Bi_2_WO_6_, except for metal oxides, various metal sulfides were used to construct a Bi_2_WO_6_-containing Z-scheme heterojunction for enhancing visible light harvesting capability, such as, Cu_2_S, MoS_2_ or MoSe_2_, SnS_2_, WS_2_, CdS, Bi_2_S_3_, In_2_S_3_, CuInS_2_, and FeIn_2_S_4_.

**Figure 6:**
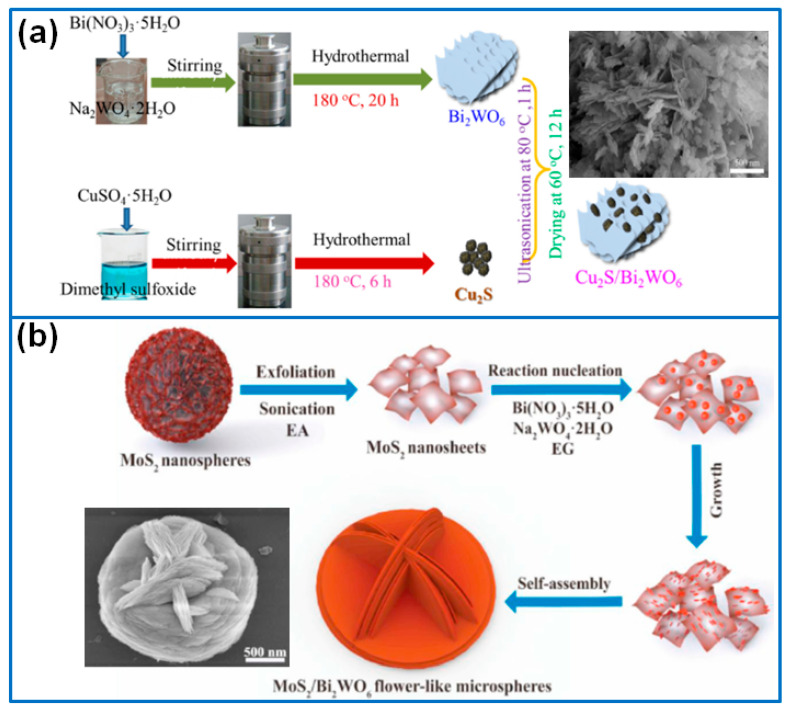
Fabrication process of (**a**) flower-like Cu_2_S/Bi_2_WO_6_ [64]. Copyright (2020) Elsevier. (**b**) MoS_2_/Bi_2_WO_6_ microspheres [65]. Copyright (2021) Elsevier.

In this field, Tang et al. [64] prepared hierarchical flower-like Cu_2_S/Bi_2_WO_6_ photocatalysts via a three-step method, in which Cu_2_S particles were distributed on the surface of Bi_2_WO_6_ nanosheets (Figure 6a). Attributed to the hierarchical structure, the enhanced visible light absorption capacity, and the Z-scheme transfer mechanism, the sample of 1%Cu_2_S/Bi_2_WO_6_ presented the highest photocatalytic activity for the removal of glyphosate. Based on the exfoliated MoS_2_ nanosheets as substrates, Zhang et al. [65] synthesized Z-scheme hetero-structured MoS_2_/Bi_2_WO_6_ hierarchical flower-like microspheres, in which the MoS_2_ substrate greatly affected the morphology and photocatalytic activity of the heterostructure (Figure 6b). The optimized composite degraded 100% Rh B within 90 min and killed almost all of Pseudomonas aeruginosa within 60 min. Similar to MoS_2_, layered MoSe_2_/Bi_2_WO_6_ composite photocatalysts were reported for the photocatalytic oxidation of gaseous toluene [66]. The formation of a p–n heterojunction provided a strong interlayer interaction, which effectively inhibited the recombination of photoinduced electron–hole pairs. The optimized 1.5%-MoSe_2_/Bi_2_WO_6_ presented the highest activity, which degraded 80% gaseous toluene within 3 h under the irradiation of visible light, and the rate constant was about 7 times and 6 times higher than that of pure MoSe_2_ and Bi_2_WO_6_, respectively.

Kumar et al. [67] synthesized a SnS_2_/Bi_2_WO_6_ plate-on-plate composite via a two-step hydrothermal route for the degradation of tetracycline (TC) and ciprofloxacin (CIP). Due to the formation of a Z-scheme heterojunction, the optimized 0.10SnS_2_/Bi_2_WO_6_ exhibited superior photocatalytic activity, which degraded 97% TC and 93% CIP within 90 min under sunlight exposure, and the degradation rate constant was three-fold higher than that of pure Bi_2_WO_6_. Similar to the plate-on-plate structure, Su et al. [68] synthesized an sTable 2D/2D WS_2_/Bi_2_WO_6_ heterostructure photocatalyst via a hydrothermal reaction. The generated Z-scheme heterostructure promoted the separation and transfer of photogenerated carriers, which showed much higher photocatalytic activity for the degradation of Rh B and OTC than pure WS_2_ or Bi_2_WO_6_.

Su et al. [69] synthesized CdS quantum dots (QDs) on a Bi_2_WO_6_ monolayer via an in situ hydrothermal method to construct a S-scheme heterojunction. The Bi–S coordination at the junction interface enhanced the charge separation and interfacial charge migration. The optimized composite containing 7% CdS exhibited the best photocatalytic activity, which completely decomposed 100 ppm C_2_H_4_ within 15 min, and the degradation rate constant was 88 times and 194 times higher than that of pure CdS and Bi_2_WO_6_. In addition, CdS nanocrystals were decorated on the surface of Bi_2_WO_6_ clusters to form CdS@Bi_2_WO_6_ photocatalysts [70]. By adjusting the microstructure of CdS, the CdS nanorod-decorated Bi_2_WO_6_ showed a higher charge separation capability than that of CdS cluster-decorated Bi_2_WO_6_, which degraded 96.1% Rh B within 120 min. Xu et al. [71] fabricated Bi_2_S_3_/2D-Bi_2_WO_6_ composite photocatalysts by using the ion exchange method, in which the Bi_2_S_3_ nanoparticle loading on Bi_2_WO_6_ nanosheets enhanced the light absorption ability and promoted the transfer and separation of photogenerated carriers. The optimized BWS-2 sample achieved the complete degradation of Rh B much better than that of Bi_2_WO_6_ nanosheets. He et al. [72] fabricated a core-shell structured In_2_S_3_/Bi_2_WO_6_ composite by using In_2_S_3_ microspheres as templates. The combination of the core and Bi_2_WO_6_ shell extended the visible-light absorption range and enabled the Z-scheme transfer pathway. Therefore, the core-shell composite achieved the fast degradation of TCH, and the activity was 2.1 and 2.4 times higher than that of pure Bi_2_WO_6_ and In_2_S_3_, respectively.

As important semiconductors, CuInS_2_ and FeIn_2_S_4_ exhibit a strong light absorption capability, with suitable energy band edges, which serve as high-performance visible light photocatalysts in the photocatalytic field. Lu et al. [73] synthesized Bi_2_WO_6_ on the surface of network-like CuInS_2_ microspheres to fabricate a Z-scheme CuInS_2_/Bi_2_WO_6_ heterojunction. The heterojunction interface enhanced the charge transfer capability, further promoting the separation of charge carriers. When used for the degradation of tetracycline hydrochloride (TCH), the activity of 15% CuInS_2_/Bi_2_WO_6_ was three-fold higher than that of pure CuInS_2_ and 17% higher than that of pure Bi_2_WO_6_. Shangguan et al. [74] prepared Z-scheme FeIn_2_S_4_/Bi_2_WO_6_ composite photocatalysts for the degradation of TCH. The formation of a direct Z-scheme heterojunction promoted the separation of photogenerated holes and electrons, which presented enhanced activity for the removal of TCH, much better than pure Bi_2_WO_6_ and FeIn_2_S_4_.

### 3.5. Bi_2_WO_6_/Bi-Series Composite

Except for Bi_2_WO_6_, other Bi-containing semiconductors, including BiOCl, BiOBr, BiOI, Bi_2_O_2_CO_3_, BiPO_4_, Bi_2_Sn_2_O_7_, BiFeO_3_, and CuBi_2_O_4_, also exhibit excellent visible light adsorption capability and photocatalytic activity. The combination of Bi_2_WO_6_ and other Bi-containing semiconductors can be regarded as an effective strategy for enhancing the photoactivity of Bi_2_WO_6_.

**Figure 7:**
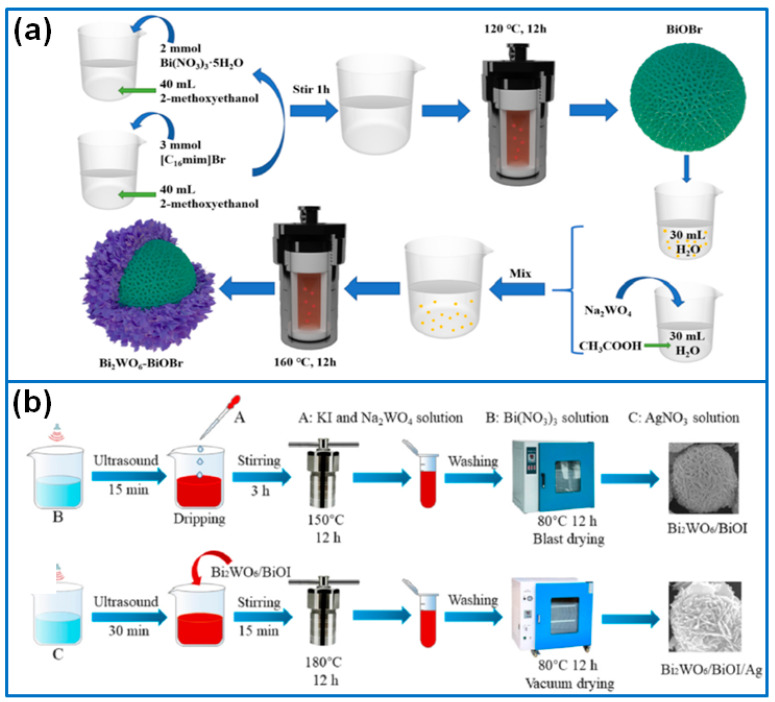
Fabrication process of (**a**) 3D/2D BiOBr/Bi_2_WO_6_ [80]. Copyright (2021) Elsevier. (**b**) Bi_2_WO_6_/BiOI/Ag heterojunction [82]. Copyright (2022) Elsevier.

In this field, Guo et al. [75] synthesized Bi_2_WO_6_ nanoparticles on layered BiOCl nanosheets to fabricate 0D/2D Bi_2_WO_6_/BiOCl composite photocatalysts. The formed heterojunction interface promoted the separation of photogenerated charge carriers. As a result, the optimized 1%Bi_2_WO_6_/BiOCl sample showed a superior degradation performance for removing OTC and phenol, and the degradation rate of OTC and phenol was 2.7-fold and 6.1-fold higher than that of pure BiOCl. Liang et al. [76] prepared a Bi_2_WO_6_/BiOCl heterojunction via the one-step hydrothermal method for the degradation of Rh B and TC. The formed heterojunction at the Bi_2_WO_6_/BiOCl interface promoted the separation of photogenerated electron–hole pairs, further improving the photocatalytic activity. Liu et al. [77] synthesized a 2D-3D BiOBr/Bi_2_WO_6_ composite with 2D Bi_2_WO_6_ nanosheets inserted in BiOBr microspheres. 3D BiOBr microspheres reduced the aggregation of Bi_2_WO_6_ nanosheets and enhanced the visible light absorption capability by providing interfacial contact. Through optimization, the BiOBr/Bi_2_WO_6_ (8:1) delivered the highest degradation efficiency for removing Rh B, TC, CIP, and MB (100%, 96%, 90% and 94%). Ren et al. [78] synthesized Bi_2_WO_6_/BiOBr composites via a one-step solvothermal route by using [C16mim] Br ionic liquid as the Br source. In this composite, Bi_2_WO_6_ nanoparticles wrapped on flower-like BiOBr and formed a type II heterojunction, which promoted the transfer and separation of charge carriers and enhanced visible light harvesting. The optimized composite with a W/Br ratio of 1:2 delivered the highest photocatalytic activity for the gradation of MB, Rh B, and TC. Chen et al. [79] synthesized a BiOBr/Bi/Bi_2_WO_6_ composite via the hydrothermal method to construct a Z-scheme heterojunction for enhancing photocatalytic activity. Due to the synergistic effect of the Z-scheme BiOBr/Bi_2_WO_6_ heterojunction and the surface plasmon resonance (SPR) effect of Bi, the optimized 20%BiOBr/7%Bi/Bi_2_WO_6_ achieved the fast degradation of Rh B under visible light, and the degradation rate of Rh B reached 98.02% within 60 min. He et al. [80] prepared a hydrangea-like BiOBr/Bi_2_WO_6_ composite via an ionic liquid-assisted hydrothermal route. The core-shell structured 3D/2D BiOBr/Bi_2_WO_6_ (Figure 7a) displayed an enhanced degradation performance for removing organic dye and drugs due to the formation of the Z-scheme heterojunction.

Besides BiOCl and BiOBr, flower-like BiOI/Bi_2_WO_6_ microspheres were prepared via the hydrothermal route for the degradation of phenol [81]. The formed heterojunction between Bi_2_WO_6_ and BiOI enhanced the separation efficiency of the electron and hole, further improving the photocatalytic activity. To further improve the photoactivity of the Bi_2_WO_6_/BiOI composite, Zheng et al. [82] deposited Ag nanoparticles onto the surface of Bi_2_WO_6_/BiOI to fabricate a Bi_2_WO_6_/BiOI/Ag heterojunction (Figure 7b). Besides the function of the heterojunction structure, Ag particles contributed to the SPR effect, which extended the visible-light absorption and accelerated the separation/transfer of photogenerated carriers. The sample of Bi_2_WO_6_/BiOI/Ag-8 displayed the highest activity for the degradation of tetracycline and a superior recycling performance.

Qiang et al. [83] synthesized I-doped Bi_2_O_2_CO_3_/Bi_2_WO_6_ heterojunction microspheres via the ionic liquid-assisted solvothermal method. The I-doped heterojunction adjusted the energy band structure and enhanced visible light adsorption, charge separation, and proton reduction. Therefore, the doped composite presented outstanding photocatalytic performance for the degradation of TC and Rh B. Wu et al. [84] prepared 3D flower-like BiPO_4_/Bi_2_WO_6_ composites via the hydrothermal method for the degradation of Rh B under visible light irradiation. The hybridization of two components accelerated the separation efficiency of charge carriers and inhibited their recombination. The composite containing 15% BiPO_4_ displayed the highest photocatalytic activity, which degraded 92% Rh B within 100 min, about 3.7 and 1.4 times higher than that of BiPO_4_ and Bi_2_WO_6_, respectively. Zhang et al. [85] deposited Bi_2_Sn_2_O_7_ (BSO) nanoparticles onto Bi_2_WO_6_ (BWO) nanosheets to fabricate flower-like Bi_2_Sn_2_O_7_/Bi_2_WO_6_ hierarchical composite photocatalysts. When used for the degradation of Rh B under visible light, the 7% BSO/BWO composite displayed the best photocatalytic activity, much better than that of pure BWO or BSO, due to the promoted separation of the photogenerated electron-hole pairs.

Tao et al. [86] synthesized Bi_2_WO_6_ nanosheets (NSs) on electrospun BiFeO_3_ nanofibers (NFs) to fabricate 1D discrete heterojunction nanofibers. The ferromagnetic feature of BiFeO_3_ facilitated the recycling treatment, and the high surface area facilitated the photocatalytic reaction by providing abundant active sites. Moreover, the 1D heterojunction promoted the separation/transport of photogenerated charges. As a result, the reaction rate constant of the nanofiber-like photocatalyst for Rh B degradation was 36.7 times and 8.7 times higher than that of pure BiFeO_3_ and Bi_2_WO_6_, respectively. Integrating the solvothermal reaction with the electrospinning technique, Teng et al. [87] prepared a one-dimensional CuBi_2_O_4_/Bi_2_WO_6_ fiber composite. Due to the formation of a Z-type heterojunction, a fiber-like photocatalyst achieved the fast degradation of more than 90% TCH within 120 min. In addition, Wang et al. [88] created flower-flake-like CuBi_2_O_4_/BWO composite via a hydrothermal route. Under the irradiation of visible light, the composite containing 60 wt% CuBi_2_O_4_ delivered the highest photocatalytic activity, which degraded 93% TC (20 mg/L) within 1 h. The superior performance was ascribed to the improved visible light absorption, interfacial charge transfer and separation, and the prolonged lifetime of photogenerated carriers.

### 3.6. Bi_2_WO_6_/Metal Tungstates Composite

Metal tungstates (MWO_4_) show the wolframite-type monoclinic structure and scheelite-type tetragonal structure, which received more attention in the photocatalytic field. The combination of Bi_2_WO_6_ with metal tungstates integrates the advantage of each component and presents enhanced photocatalytic activity for the degradation of organic pollutants. In this field, Kumar et al. [89] synthesized a Bi_2_WO_6_/ZnWO_4_ composite photocatalyst via the modified hydrothermal method. The bi-crystalline framework of Bi_2_WO_6_ and ZnWO_4_ played a synergistic effect, which reduced the crystallite size and band gap and effectively separated and transferred the photo-generated electron–hole pairs. Under UV irradiation, the optimized 30% Bi_2_WO_6_/ZnWO_4_ delivered the maximum degradation performance for the removal of Plasmocorinth B dye. Miao et al. [90] prepared Sb_2_WO_6_/Bi_2_WO_6_ composite photocatalysts and evaluated their photocatalytic activity for the degradation of Rh B and MO. The composite showed an increased specific surface area and an enhanced visible-light absorption capability, and it suppressed the recombination of electron–hole pairs. The composite containing 6% Sb achieved the fast degradation of 100% Rh B and 70% MO within 90 min, much better than pure single phase Bi_2_WO_6_ and Sb_2_WO_6_. Ni et al. [91] synthesized a flower-like Ag_2_WO_4_/Bi_2_WO_6_ (AWO/BWO) composite for the degradation of Rh B. AWO and BWO formed a direct Z-scheme heterojunction, which promoted the migration of interface charges, enhanced the light absorption capability, and inhibited the recombination of the electron-hole pairs. The composite containing 3 wt% AWO exhibited the highest activity, which degraded nearly 100% Rh B within 150 min, 11.5-fold and 1.5-fold higher than that of pristine AWO and BWO.

## 4. Bi_2_WO_6_-Based Ternary Composite

Besides Bi_2_WO_6_-based binary heterojunction composites, lots of Bi_2_WO_6_-containing ternary composites were developed as high-performance photocatalysts. From the reported Bi_2_WO_6_-based ternary composites, commonly used components involve carbon materials, g-C_3_N_4_, BiOX, AgBr, Ag_2_CO_3_, Ag_2_O, Cu_2_O, TiO_2_, ZnO, Ti_3_C_2_, Bi_2_MoO_6_, BiPO_4_, and Au/Ag nanoparticles. Compared to binary composites, the advantage of ternary composites exhibits optimized light harvesting capability and photocatalytic activity due to the constructed double heterostructure interfaces and the synergistic effect derived from three components. In view of the broad selectivity of semiconductor candidates, it is hard to summarize the design principle of Bi_2_WO_6_-based ternary composites. However, the fixed binary combination of Bi_2_WO_6_/g-C_3_N_4_, Bi_2_WO_6_/carbon materials, Bi_2_WO_6_/Au or Ag-based materials, and Bi_2_WO_6_/Bi-series semiconductors is reported to hybridize with the third component.

### 4.1. The Composite of Bi_2_WO_6_/g-C_3_N_4_/Other Materials

In respect of the Bi_2_WO_6_/g-C_3_N_4_ combination, Zhang et al. [92] prepared a dual Z-scheme BiSI/Bi_2_WO_6_/g-C_3_N_4_ photocatalyst via hydrothermal method. The generation of dual Z-scheme heterojunction promoted the transfer and separation of photogenerated electron–hole pairs. When used for the degradation of TC, Rh B, and chlortetracycline (CTC), the optimized BiSI/Bi_2_WO_6_/20%g-C_3_N_4_ exhibited the highest photocatalytic activity, much better than that of the single and binary systems. Sun et al. [93] fabricated a double Z-scheme g-C_3_N_4_/Bi_2_MoO_6_/Bi_2_WO_6_ (CN/MO/WO) composite for the degradation of TC. In this ternary system, g-C_3_N_4_ enhanced the specific surface area and accelerated the carrier transfer. Bi_2_WO_6_ and Bi_2_MoO_6_ extended the light absorption range and inhibited the recombination of photogenerated electron–hole pairs. As a result, the optimized 15% CN/MO/WO composite achieved the fast photodegradation of 98% TC within 30 min under the irradiation of visible light. Zhou et al. [94] synthesized dual Z-scheme BiOBr/g-C_3_N_4_/Bi_2_WO_6_ photocatalysts via one-pot hydrothermal reaction. The dual heterojunction effectively suppressed the recombination of photogenerated carriers and presented superior photocatalytic activity, which degraded 90% TC within 40 min under the irradiation of visible light. In addition, a Bi_2_WO_6_/BiOI/g-C_3_N_4_ ternary composite photocatalyst was prepared for the degradation of TC [95], and the optimized composite degraded over 90% TC within 120 min. Moreover, the ternary photocatalyst also exhibited a superior performance for the degradation of municipal waste transfer station leachate.

**Figure 8:**
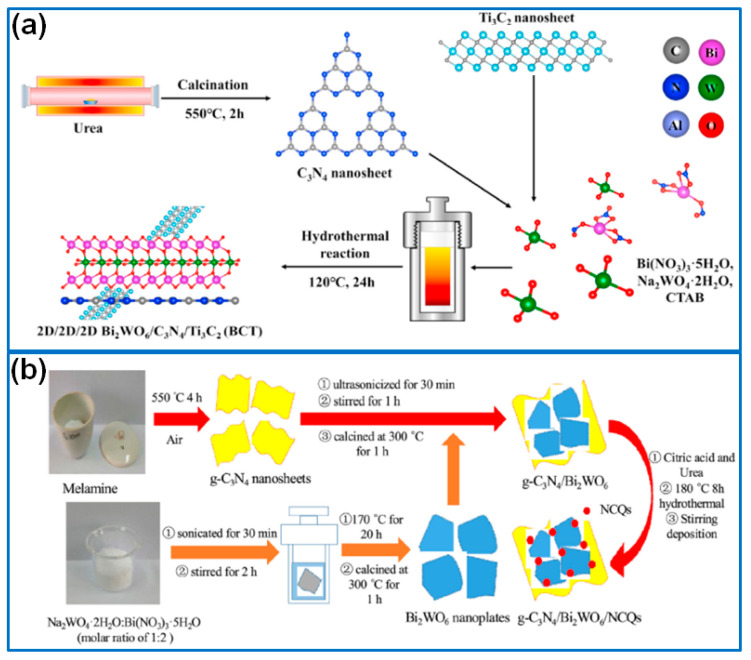
Preparation of (**a**) Bi_2_WO_6_/g-C_3_N_4_/Ti_3_C_2_ [97]. Copyright (2020) Elsevier. (**b**) g-C_3_N_4_/Bi_2_WO_6_/NCQs ternary composite [99]. Copyright (2020) Elsevier.

Hu et al. [96] fabricated a ternary heterojunction g-C_3_N_4_/BiVO_4_-Bi_2_WO_6_ photocatalyst by the intercalation of a BiVO_4_-Bi_2_WO_6_ composite into compressed layered g-C_3_N_4_ nanosheets. The compressed layer structure accelerated the transfer of electrons and the generation of superoxide radicals, which enhanced photocatalytic activity, and the degradation efficiency of Rh B and TC was 96.7% and 94.8% within 60 min, respectively. In another work, a 2D/2D/2D Bi_2_WO_6_/g-C_3_N_4_/Ti_3_C_2_ composite (Figure 8a) was prepared via a one-step hydrothermal reaction [97]. In this system, seamless interfacial contact of the 2D heterojunction facilitated the separation and transfer of photogenerated electron–hole pairs. Moreover, Ti_3_C_2_ also promoted charge separation. As a result, the composite achieved the fast photodegradation of CIP, and the reaction rate constant was 4.78 times higher than that of Bi_2_WO_6_. Li et al. [98] prepared a 2D/2D Z-scheme g-C_3_N_4_/Au/Bi_2_WO_6_ (CN/Au/BWO) composite for the photodegradation of Rh B. Serving as a redox mediator, Au nanoparticles accelerated the transmission and separation of photogenerated carriers. Moreover, the 2D/2D Z-scheme structure provided abundant active sites for enhancing the photocatalytic activity. The CN/Au(1)/BWO sample degraded 88.7% Rh B within 30 min, and the rate constant was 1.48-fold and 1.62-fold higher than that of pure BWO and CN, respectively. To further enhance the photocatalytic activity of the g-C_3_N_4_/Bi_2_WO_6_ Z-scheme heterojunction, Jia et al. [99] introduced nitrogen-doped carbon quantum dots (NCQs) onto a g-C_3_N_4_/Bi_2_WO_6_ interface to form g-C_3_N_4_/Bi_2_WO_6_/NCQs ternary composites (Figure 8b). The NCQs extended the light absorption range and promoted the transfer and separation of photogenerated electron–hole pairs. Compared to single or binary composites, the ternary composite showed the highest degradation efficiency for the removal of Rh B and TC under visible light irradiation.

### 4.2. The Composite of Bi_2_WO_6_/Carbon/Other Materials

In the field of Bi_2_WO_6_/carbon materials, Guan et al. [100] synthesized a ternary AgBr/GO/Bi_2_WO_6_ Z-scheme photocatalyst and discussed the effect of AgBr and GO fractions on photocatalytic activity. The optimized 15%AgBr/5GO/Bi_2_WO_6_ delivered the highest degradation efficiency for the removal of 84% TC under visible light, and the reaction kinetic constant was about 3.16-fold and 4.60-fold higher than that of pure Bi_2_WO_6_ and AgBr, respectively. The superior performance was due to an extended visible light adsorption range and an enhanced charge separation and transfer. Zhu et al. [101] prepared a GO@BiOI/Bi_2_WO_6_ composite for the removal of Bisphenol A (BPA). In this ternary system, GO effectively modified the surface of BiOI/Bi_2_WO_6_ and improved the physico-chemical property. The optimized composite degraded 81% BPA within 5 h under the irradiation of UV-vis light. Tian et al. [102] prepared a Z-scheme flower-like Bi_2_MoO_6_/Bi_2_WO_6_/MWCNTs photocatalyst via hydrothermal route. Under visible light irradiation, the ternary composite degraded 96% reactive blue 19 (RB-19) within 4 h, the photocatalytic efficiency was much higher than that of Bi_2_MoO_6_/MWCNTs, and pure Bi_2_MoO_6_ and Bi_2_WO_6_. Niu et al. [103] synthesized Bi_2_WO_6_/C@Cu_2_O Z-scheme photocatalysts for TC degradation. The wrapped carbon layer on Cu_2_O avoided the photo-corrosion of Cu_2_O. Furthermore, the oxygen-containing groups in the carbon layer decreased interfacial resistance and promoted electron transfer. The degradation rate constant of the ternary composite was 2.8 times higher than that of pure Bi_2_WO_6_.

### 4.3. The Composite of Bi_2_WO_6_/Au or Ag-Based Materials/Other Materials

Among the Bi_2_WO_6_/Ag-based materials, Wang et al. [104] prepared a Ag_2_CO_3_/AgBr/Bi_2_WO_6_ ternary photocatalyst via a precipitation method. When used for the degradation of Rh B, the degradation rate of the ternary composite was 95.1% within 60 min under solar illumination, and the degradation efficiency was much higher than that of each component. Gang et al. [105] synthesized a Ag/AgBr/Bi_2_WO_6_ composite via the oil/water self-assembly method. In this ternary composite, Ag/AgBr was uniformly dispersed on the Bi_2_WO_6_ surface, which extended the visible-light absorption range for the surface plasmonic resonance (SPR) effect of Ag. Moreover, the composite accelerated the separation of photogenerated charges. When utilized for the degradation of Rh B and phenol, the ternary composite presented optimum photocatalytic activity under visible light, much better than Ag/AgBr and pure Bi_2_WO_6_. Jin et al. [106] prepared a Au@TiO_2_/Bi_2_WO_6_ composite via a sol-gel method followed by hydrothermal reaction. In this ternary system, core-shell structured Au@TiO_2_ nanoparticles were dispersed on flower-like Bi_2_WO_6_ nanosheets. The formation of a Z-scheme heterojunction and SPR effect of Au promoted the generation, separation, and interfacial transfer of photogenerated charge carriers. When served for the degradation of sulfamethoxazole (SMX) and TCH under visible light, the degradation rate was 96.9% and 95.0% within 75 min, respectively. Moreover, the degradation rate constant was 7.2 times and 1.9 times higher than that of pure Bi_2_WO_6_, respectively.

### 4.4. The Composite of Bi_2_WO_6_/Bi-Series Semiconductors/Other Materials

In the field of Bi_2_WO_6_/Bi-series semiconductors, Zhu et al. [107] prepared a magnetic Bi_2_WO_6_/BiOI@Fe_3_O_4_ ternary composite for the photodegradation of TC. The optimized Bi_2_WO_6_/BiOI@5%Fe_3_O_4_ sample showed the highest TC degradation rate of 97%, much higher than that of pure Bi_2_WO_6_ (63%). Moreover, the spent powder can be magnetically recycled, and the recycled sample also exhibited good photocatalytic activity. Combining the electrostatic spinning technique, Chen et al. [108] fabricated 1D magnetic flower-like CoFe_2_O_4_@Bi_2_WO_6_@BiOBr photocatalysts for the degradation of Rh B. The resulting flower-like heterojunction enhanced the specific surface area and accelerated the separation of photogenerated charge carriers. Consequently, the ternary composite degraded 92.08% Rh B within 3 h.

### 4.5. Other Composites

In view of the high surface area and tight interfacial contact of 2D nanomaterials [109,110,111], Sharma et al. [112] prepared a 2D-2D-2D ZnO/Bi_2_WO_6_/Ti_3_C_2_ ternary composite photocatalyst via two-step electrostatic assembly. The optimized ZBT05 containing 5 wt% Ti_3_C_2_ delivered the highest degradation rate (~77%) for the removal of ciprofloxacin (CFX) within 160 min due to the enhanced photogenerated charge carrier separation caused by the generated ternary interface. Besides ternary composites, the composite photocatalysts composed of four or five components were reported for enhancing photocatalytic activity. In this respect, Ma et al. [113] prepared a GO-modified Ag/Ag_2_O/BiPO_4_/Bi_2_WO_6_ multi-component composite photocatalyst and investigated the photocatalytic activity for the degradation of Rh B and amoxicillin (AMX). The composite exhibited a small size, fast charge transfer efficiency, and extended light absorption range, which presented enhanced photocatalytic activity for the degradation of AMX, Rh B, and E. coli under visible light irradiation.

## 5. Conclusions and Prospects

To sum up, the advances of Bi_2_WO_6_-based photocatalysts are summarized in this review, including morphology control, the surface modification and heteroatom doping of Bi_2_WO_6_, Bi_2_WO_6_-based binary composites, and Bi_2_WO_6_-based ternary composites. The most popular synthesis method of Bi_2_WO_6_ is the hydrothermal or solvothermal method, and the reaction temperature and time heavily affect the microstructure and photocatalytic performance of Bi_2_WO_6_. The surfactants of CTAB and PVP were used to adjust the microstructure of Bi_2_WO_6_. Furthermore, Au-decorated Bi_2_WO_6_ hollow microspheres were synthesized to utilize the SPR effect of Au nanoparticles. Heteroatom doping can be used to enhance the photoactivity of Bi_2_WO_6_. Among various dopants, N, F, Cl, and I serve as non-metal dopants for doping Bi_2_WO_6_. In addition, Fe, Ti, Sr, Er, La, Au, Ag, and Mo are used to fabricate metal-doped Bi_2_WO_6_. Besides single atom doping, (La, Mo) co-doped Bi_2_WO_6_ was reported to enhance the photoactivity of Bi_2_WO_6_ by adjusting the particle size and lattice spacing. In view of the limited photocatalytic activity of single Bi_2_WO_6_, the development of Bi_2_WO_6_-based binary and ternary composites has become a major topic for constructing high-performance photocatalysts. Bi_2_WO_6_-based binary composites show a wide research range for the diversity of alternative materials. The existing Bi_2_WO_6_-based binary composites can be classified into six types: Bi_2_WO_6_/carbon or MOF composite, Bi_2_WO_6_/g-C_3_N_4_ composite, Bi_2_WO_6_/metal oxides composite, Bi_2_WO_6_/metal sulfides composite, Bi_2_WO_6_/Bi-series composite, and Bi_2_WO_6_/metal tungstates composite. Due to the diversity of target organic pollutants, and the difference of pollutant concentration, light source or powder, and catalyst dosage, it is very difficult to compare the photocatalytic activity of different Bi_2_WO_6_-based binary composites. Compared to other semiconductors, g-C_3_N_4_ and metal oxides are widely used to hybridize with Bi_2_WO_6_, and the resulting Bi_2_WO_6_/g-C_3_N_4_ and Bi_2_WO_6_/metal oxides composites deliver enhanced photodegradation efficiency, which is much better than each component. Besides Bi_2_WO_6_-based binary composites, lots of Bi_2_WO_6_-based ternary composites were developed as high-performance photocatalysts. The commonly used components include carbon materials, g-C_3_N_4_, BiOX, AgBr, Ag_2_CO_3_, Ag_2_O, Cu_2_O, TiO_2_, ZnO, Ti_3_C_2_, Bi_2_MoO_6_, BiPO_4_, and Au/Ag nanoparticles. According to the material type, binary Bi_2_WO_6_/g-C_3_N_4_, Bi_2_WO_6_/carbon materials, Bi_2_WO_6_/Au or Ag-based materials, and Bi_2_WO_6_/Bi-series semiconductors were fabricated for further hybridizing with the third component, and they present outstanding photocatalytic activity for the formation of double heterostructures and the synergistic effect of three components. In addition, a GO modified Ag/Ag_2_O/BiPO_4_/Bi_2_WO_6_ multi-component composite was synthesized to further improve photocatalytic activity.

Based on the summary above, abundant progress has been achieved in Bi_2_WO_6_-based photocatalysts. However, some urgent problems still exist, such as the controllable microstructure, the suitable component and ratio optimization, and the photocatalytic mechanism of different Bi_2_WO_6_-based composites. Aiming to solving the three problems mentioned above, we put forward the following promising research trends:

(1) The controllable synthesis and microstructure optimization of Bi_2_WO_6_ and Bi_2_WO_6_-based composite. The ideal microstructures of photocatalysts include hierarchical hollow structures, flowers, or spheres with a high specific surface area. Moreover, binary or ternary composites should have a strong interfacial binding strength, and the ratio optimization of different components is a major task.
(2) The selection of suitable candidate semiconductor photocatalysts. The selection of semiconductors should consider the band gap feature of Bi_2_WO_6_, and the resulting Bi_2_WO_6_-based composite should form a Z-scheme, S-scheme heterojunction, or double heterojunctions. In addition, the heteroatom doping and introduction of noble metal nanoparticles can be adopted as an effective strategy for enhancing photocatalytic activity.
(3) The combination of theoretical calculation and experimental results clarify the photocatalytic mechanism. The photocatalytic mechanism of the Bi_2_WO_6_-based composite is the difficulty for designing high-performance hybrid photocatalysts. Besides the traditional characterization techniques, theory computations should be paid more attention for clarifying the photocatalytic mechanism.
